# Xenobiotic Toxicants and Particulate Matter: Effects, Mechanisms, Impacts on Human Health, and Mitigation Strategies

**DOI:** 10.3390/jox15040131

**Published:** 2025-08-14

**Authors:** Tamara Lang, Anna-Maria Lipp, Christian Wechselberger

**Affiliations:** 1 Department of Pathophysiology, Institute of Physiology and Pathophysiology, Medical Faculty, Johannes Kepler University Linz, 4020 Linz, Austria; tamara.lang@jku.at; 2 Clinical Research Institute for Cardiovascular and Metabolic Diseases, Medical Faculty, Johannes Kepler University Linz, 4020 Linz, Austria; 3 Core Facility Cytometry, Center for Medical Research, Medical Faculty, Johannes Kepler University Linz, 4020 Linz, Austria; anna-maria.lipp@jku.at

**Keywords:** particulate matter, respiratory disease, cardiovascular disease, neurological effects, microbiome, mitigation strategies

## Abstract

Particulate matter (PM), a complex mixture of solid particles and liquid droplets, originates from both natural sources, such as sand, pollen, and marine salts, and anthropogenic activities, including vehicle emissions and industrial processes. While PM itself is not inherently toxic in all its forms, it often acts as a carrier of xenobiotic toxicants, such as heavy metals and organic pollutants, which adhere to its surface. This combination can result in synergistic toxic effects, significantly enhancing the potential harm to biological systems. Due to its small size and composition, PM can penetrate deep into the respiratory tract, acting as a physical “shuttle” that facilitates the distribution and bioavailability of toxic substances to distant organs. The omnipresence of PM in the environment leads to unavoidable and constant exposure, contributing to increased morbidity and mortality rates, particularly among vulnerable populations like the elderly, children, and individuals with pre-existing health conditions. This exposure also imposes a substantial financial burden on healthcare systems, as treating PM-related illnesses requires significant medical resources and leads to higher healthcare costs. Addressing these challenges necessitates effective mitigation strategies, including reducing PM exposure, improving air quality, and exploring novel approaches such as AI-based exposure prediction and nutritional interventions to protect public health and minimize the adverse effects of PM pollution.

## 1. Introduction

Particulate matter (PM) refers to a mixture of solid particles and liquid droplets found in the air, which vary in size and composition. PM is typically classified based on its aerodynamic diameter into categories such as PM_10_ (particles with diameters less than 10 micrometers), PM_2.5_ (particles with diameters less than 2.5 μm), and ultrafine particles (UFPs; particles with diameters less than 0.1 μm) [1]. PM in the atmosphere originates from both natural sources, such as dust storms, wildfires, and volcanic eruptions, and anthropogenic sources, including vehicle emissions, industrial processes, and construction activities [2,3,4,5].

Xenobiotic toxicants can cause harmful biological responses in the human body, which range from immunotoxicity and mitochondrial dysfunction to genotoxicity, organ damage, or cancer depending on the type, dose and duration of exposure [6]. The presence of toxic xenobiotics on the surface of PM can exacerbate the harmful effects on human health. This occurs because PM increases the bioavailability of xenobiotics by providing a larger surface area and acting as a carrier, enabling the delivery of these toxic substances deep into the respiratory system, where they can enter the bloodstream and reach various organs, causing systemic damage. Additionally, xenobiotic toxicants can amplify the inflammatory and oxidative effects of PM, resulting in more severe health outcomes. Understanding the effects of PM loaded with xenobiotic toxicants is therefore crucial for medical research, as exposure can convincingly be linked to various adverse health outcomes, including respiratory, neurological and cardiovascular diseases, and recent studies have already highlighted the importance of investigating these effects to develop effective public health strategies [7,8,9]. The chemical composition of environmental and industrial PM is complex and varies significantly. Characterizing the chemical composition and sources is therefore essential for assessing its potential impacts on human health [10]. Toxic xenobiotic substances, on the other hand, also include a broad spectrum of representatives (Table 1). For example, organic compounds can include polycyclic aromatic hydrocarbons (PAHs) and volatile organic compounds (VOCs), while heavy metals such as Pb, Hg, and Cd are often found in PM from industrial emissions [11,12,13]. Inorganic salts, including sulfates, nitrates, and ammonium, are commonly present in PM from both natural and human activities [14,15]. This review explores the toxic mechanisms of particulate matter (PM) containing xenobiotic toxicants to better understand their role in adverse health outcomes. Given that ambient PM is associated with 3–4 million deaths annually (approximately 7.6% of global mortality) and 103.1 million disability-adjusted life years (4.2% globally) [16], effective public health strategies and mitigation initiatives are also provided.

## 2. Environmental PM

**Figure 1:**
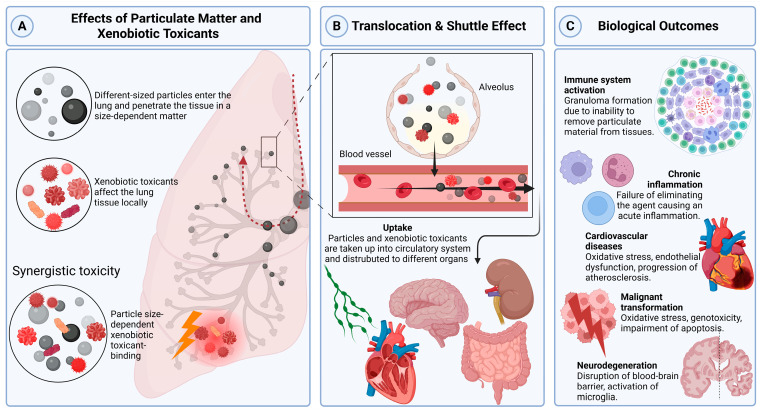
Schematic representation of the uptake, systemic distribution, and health impacts of particulate matter (PM) based on the particle size. (**A**) Fine (PM_2.5_) and ultrafine particles (PM_0.1_) are inhaled into the lungs, where their size determines their deposition and interaction with lung tissue. Larger particles are primarily deposited in the upper airways, while smaller particles penetrate deeper into the alveoli. (**B**) Ultrafine particles can translocate across the alveolar–capillary barrier into the bloodstream, enabling systemic distribution to distant organs. The toxin-binding capacity of particles increases with a decreasing size, as smaller particles have a larger surface area-to-volume ratio, allowing them to carry more adsorbed toxic substances. (**C**) Systemic circulation of particles leads to inflammation, oxidative stress, and tissue damage in multiple organs, including the brain (neurodegeneration) and the heart (cardiovascular disease). Chronic exposure to particulate matter is associated with adverse health outcomes, including respiratory diseases, cardiovascular disorders, neurodegenerative diseases, and malignant transformation of cells, ultimately leading to cancer. (Created in BioRender. Reingruber, S. (2025), https://BioRender.com/5rrwxa2, accessed on 11 August 2025).

Environmental PM exerts its effects through a complex chain of events. The first critical factor is the pathway of exposure, as the entry route strongly influences whether xenobiotic toxicants act locally or are transported systemically. Xenobiotic toxicants attached to PM can exert immediate toxic effects at the primary site of contact (e.g., lung tissue), whereas PM particles themselves can translocate across biological barriers and act as shuttles, distributing adsorbed toxicants to distant organs. These physicochemical interactions determine the particles’ bioavailability and capacity to cross biological barriers, ultimately dictating their tissue distribution. Once deposited in the target organs, PM and its associated toxicants can trigger immune responses, chronic inflammation, granuloma formation, and long-term disease outcomes (Figure 1).

The following subsections describe this process stepwise, starting with the exposure pathways to highlight their role in shaping the fate and toxicity of PM-associated substances, and then linking the particle–xenobiotic interactions, systemic distribution, and pathophysiological effects. Unlike previous reviews that considered PM and toxicants separately, this review specifically examines PM as an active carrier of xenobiotic substances, highlighting how these interactions shape the systemic distribution and amplify the toxicity.

### 2.1. Pathways of Human Exposure to PM

PM can enter the human body through various routes of exposure, each associated with specific health concerns [31]. Inhalation is the primary route, with minor contributions from ingestion, ocular exposure, and dermal absorption [37,38,39].

Upon inhalation, PM is deposited along the respiratory tract in a size-dependent manner: coarse particles (PM_10_, ≤10 μm) settle in the upper airways (e.g., nasal cavity, trachea), fine particles (PM_2.5_, ≤2.5 μm) reach the bronchi and alveoli, and UFPs (PM_0.1_, ≤0.1 μm) can translocate into the bloodstream [40]. Once in circulation, UFPs may affect extrapulmonary organs such as the liver, spleen, heart, and kidneys, or even bypass the blood–brain barrier via the olfactory nerve [37,41,42,43,44]. As a protective mechanism, PM and its soluble components are primarily cleared by mucociliary mechanisms and renal excretion [45,46].

PM is able to interact with various cell types in the lungs, including epithelial cells and macrophages, triggering immune responses and inflammation [47,48]. Smaller PM fractions, particularly UFPs, pose increased health risks due to their higher particle number and greater surface area at the same mass concentration [49]. This facilitates stronger interactions with biological membranes and enhanced uptake. Whereas uptake of coarse and fine PM is generally mediated by macrophages through receptor- and actin-dependent mechanisms, UFPs are internalized by non-specific pathways that also allow entry into other cell types, such as erythrocytes [50].

Beyond the lungs, ingestion represents another route of exposure, where contaminated food or water can introduce PM into the gastrointestinal tract [51,52,53,54]. Ingested PM with or without the associated xenobiotic toxicants can affect cells of the gastrointestinal tract, leading to inflammation and more severe issues like cancer development [55,56,57]. Dermal exposure, though less common, can occur via direct skin contact with airborne particles or contaminated surfaces, potentially leading to skin irritation, allergic reactions, and barrier disruption [57,58,59]. Ocular exposure is also increasingly recognized as a relevant route, where PM can contact the ocular surface, leading to oxidative stress, inflammation, and symptoms consistent with dry eye disease, as shown in both animal models and human studies [39,60,61].

Satellite-based monitoring of PM has emerged as a critical tool for assessing air quality on the regional and global scales, offering extensive spatial coverage that complements traditional ground-based monitoring networks. Current methods leverage satellite sensors, such as Moderate Resolution Imaging Spectroradiometer (MODIS), Multi-angle Imaging SpectroRadiometer (MISR), and Visible Infrared Imaging Radiometer Suite (VIIRS), to estimate the aerosol optical depth (AOD), which serves as a proxy for the PM concentration [62]. However, the resolution of satellite data, both spatial and temporal, remains a key consideration. High-resolution sensors, such as those on Sentinel-5P and upcoming missions like Tropospheric Emissions: Monitoring of Pollution (TEMPO), are improving the ability to capture fine-scale variations in PM levels [63]. Validation of satellite-derived PM estimates is essential to ensure the accuracy and reliability, typically achieved through comparison with ground-based measurements from networks like the Aerosol Robotic Network (AERONET) and regulatory air quality monitoring stations [64,65,66,67]. Data fusion techniques, such as machine learning models, geostatistical methods, and chemical transport models, are increasingly employed to integrate satellite data with ground-level observations, enhancing the spatial and temporal resolution of PM estimates [68]. These approaches enable the generation of high-quality, near-real-time PM datasets that are critical for epidemiological studies, exposure assessments, and policy-making. Despite these advancements, challenges remain, including accounting for the vertical aerosol distribution, cloud cover interference, and the need for region-specific calibration to address local atmospheric conditions. Continued innovation in satellite technology, combined with robust validation and integration frameworks, holds significant promise for advancing PM monitoring and its applications in public health and environmental management.

### 2.2. Interaction Between PM and Toxic Substances

The size of particles from environmental sources is inversely related to their surface area-to-volume ratio. As the particle size decreases, the surface area relative to the volume increases significantly, thus providing more active sites for interactions and making them highly effective at binding (e.g., hydrophobic drugs) [69,70]. This property is particularly relevant in environmental and biological contexts, where smaller particles can also act as carriers of xenobiotic and toxic compounds, influencing their transport and bioavailability. The combined impact of PM-induced nonspecific stress responses and the disruption of physiological processes at the cellular level can ultimately result in systemic inflammation and oxidative stress, which are further intensified and amplified by the biological activity of the xenobiotic toxicants [71]. This can facilitate the development of cardiovascular diseases, including heart attacks and hypertension, as well as the induction of neurological effects like cognitive decline and neurodegenerative diseases [7,72,73,74]. Furthermore, PM exposure can disrupt endocrine function and affect reproductive health, leading to serious issues such as birth defects and low birth weight [75,76,77].

The health impacts of PM exposure are not uniformly distributed across populations, with certain groups experiencing heightened vulnerability due to a combination of biological, social, and environmental factors. Vulnerable populations, such as children, the elderly, individuals with pre-existing health conditions, and those from lower socioeconomic backgrounds, are disproportionately affected by PM exposure. Exploring the interplay between PM exposure and sociodemographic factors requires a life-course perspective, which considers how cumulative exposures and critical windows of susceptibility (e.g., during early development or aging) influence health outcomes [78,79]. For instance, prenatal and early-life exposure to PM has been linked to adverse developmental outcomes, while chronic exposure in later life exacerbates cardiovascular and respiratory diseases [80,81,82]. Incorporating the theme of environmental justice is essential, as marginalized communities often face higher PM exposure due to proximity to pollution sources, such as industrial facilities, highways, and densely populated urban areas, while also having limited access to healthcare and resources to mitigate exposure. Health disparities are further amplified by systemic inequities, including housing quality, occupational exposures, and access to clean air. To address these issues, research should integrate sociodemographic data with PM exposure assessments using tools like geographic information systems (GISs), land-use regression models, and exposome frameworks [83,84,85,86]. Additionally, studies should examine the intersection of PM exposure with other social determinants of health, such as race, income, and education, to identify at-risk populations and inform targeted interventions. By adopting an interdisciplinary approach that combines environmental science, epidemiology, and social justice, researchers can better understand and address the inequities in PM-related health outcomes, ultimately guiding policies that promote environmental equity and public health.

The toxicokinetics and toxicodynamics of PM are strongly influenced by its chemical composition, particularly when it carries adsorbed xenobiotic and toxic substances such as metals, PAHs, and endotoxins [87]. These substances can modulate cellular responses, increase bioavailability, and exacerbate toxicity (Table 2). Some compounds may even dissociate from the particle core, entering systemic circulation and exhibiting independent toxicological profiles.

As shown in Table 3, xenobiotic toxicants can interact in various ways, with PM acting as a carrier of and delivery system for these associated toxicants, enhancing their distribution to target tissues and intensifying their biological effects. Health outcomes are further modulated by individual susceptibility, co-exposure to other pollutants, and overall exposure levels [89]. Recent advances in environmental toxicology have led to the development of integrated toxicokinetic/toxicodynamic (TK/TD) models, which simulate the PM deposition, translocation, and cellular effects over time. These models are critical for improving risk prediction and refining public health assessment strategies [16,88,99].

### 2.3. Localized and Systemic Effects of PM

Genuine xenobiotic and toxic substances often exert their toxicity directly or at least close to the site of contact since this is the area where the highest concentration of the substances is present [114]. Systemic effects, on the other hand, often need higher loads of contamination and/or longer times of exposure to the respective substances [115]. On the other hand, subsequent effects exerted through PM in combination with xenobiotic toxicants are often more profound, since translocation from the initial site of deposition to other parts of the body is relatively easy depending on the size of the particles and can occur, for example, with the help of phagocytic immune cells [116]. Upon entering the bloodstream, PM can disseminate to vital organs such as the heart, brain, and kidneys. In response, macrophages and other immune cells attempt to engulf and neutralize the particles, often resulting in the formation of granulomas—organized collections of immune cells and epithelial cells that encapsulate foreign materials to isolate them from the surrounding tissue, often associated with chronic inflammation [117,118,119,120]. The release of specific inflammatory cytokines and chemokines can later on cause tissue damage and remodeling [121]. The tissues and organs most commonly affected by granuloma formation due to the accumulation of such particulate matter include, but are not limited to, lungs, the lymph nodes and skin [122]. In addition, tissues and organs rich in capillary structures, such as the liver, spleen and kidneys, can develop granulomas in response to embedded foreign particles [122]. In general, the severity and location of granuloma formation depends on the type of particulate matter, the route of exposure, and the individual’s immune response [123]. Complications associated with granuloma formation range from impaired organ function to chronic inflammation and fibrosis [124]. Also, an increased risk of cancer development has already been associated with PM-induced granulomas, underscoring the relevance of this area of research [125,126].

### 2.4. Emerging Pollutants

Emerging pollutants such as nanoplastics, fullerenes, and other organic and inorganic nanoparticles pose significant risks to public health due to their unique physicochemical properties, including their small size, high surface area, and potential for bioaccumulation [127,128]. These particles can penetrate biological barriers, such as the respiratory and gastrointestinal tracts, and even cross the blood–brain barrier, leading to oxidative stress, inflammation, and cellular damage [129]. Nanoplastics, for instance, can adsorb toxic chemicals and heavy metals, acting as carriers that amplify their harmful effects [130,131]. Similarly, engineered nanoparticles like fullerenes may generate ROS, contributing to chronic diseases such as cancer, cardiovascular disorders, and neurodegenerative conditions [132,133]. The long-term impacts of these pollutants remain poorly understood, but their persistence in the environment and potential for widespread exposure underscore the urgent need for research and regulatory measures to mitigate their effects on human health and ecosystems.

## 3. Health Impacts of PM Exposure

### 3.1. Role of Oxidative Stress and Inflammation

One of the primary mechanisms by which PM exerts its harmful effects is through oxidative stress and inflammation [134]. This oxidative stress can trigger inflammatory responses, leading to the release of cytokines and other inflammatory mediators, and numerous studies have already demonstrated the role of oxidative stress and inflammation in PM-induced health effects [135,136,137]. The induction of oxidative stress occurs through several mechanisms once PM becomes deposited in the respiratory tract and interacts with, for example, epithelial cells and macrophages [138,139]. This effect is exacerbated if PM contains additional components, such as heavy metals and organic compounds, which can directly generate reactive oxygen species (ROS) or disrupt cellular antioxidant defenses [140]. Due to the presence of transition metals like Fe and Cu, ROS formation can be catalyzed through Fenton reactions [141]. Additionally, organic compounds in PM, such as PAHs, can undergo metabolic activation to produce ROS [142]. The excessive production of ROS ultimately overwhelms the cellular antioxidant capacity, leading to oxidative damage to lipids, proteins, and DNA. This oxidative stress therefore represents a key mechanism underlying the adverse health effects of PM exposure, including respiratory and cardiovascular diseases [143].

PM exposure can lead to the induction of inflammation in human cells and tissues through multiple pathways [144,145]. When PM is inhaled, it can activate epithelial cells and alveolar macrophages in the respiratory tract, triggering the release of pro-inflammatory cytokines such as interleukin-6 (IL-6), tumor necrosis factor-alpha (TNF-α), and interleukin-1 beta (IL-1β), which recruit and activate additional immune cells, including neutrophils and lymphocytes, amplifying the inflammatory response [146,147,148]. PM components, such as endotoxins and organic compounds, can furthermore interact with toll-like receptors (TLRs) on immune cells, further promoting inflammation [149,150]. Such a chronic activation of inflammatory pathways can ultimately lead to tissue damage and contribute to the development of diseases such as asthma, chronic obstructive pulmonary disease (COPD), and cardiovascular conditions [151,152]. Recent studies indicate that PM loaded with xenobiotic toxicants induces prolonged oxidative stress compared to exposure to particles alone, highlighting a synergistic effect that has been receiving growing scientific attention in the past few years [153,154,155].

### 3.2. Immune System Effects and Allergic Reactions to PM

PM also exerts significant modulatory effects on different cell types of the immune system, which vary with the particle size, concentration, chemical composition, and exposure duration (Table 4). These effects, both immunostimulatory and immunosuppressive, contribute to a spectrum of diseases, including respiratory, cardiovascular, autoimmune, and systemic inflammatory conditions [37,156,157]. Key PM constituents, such as metals, PAHs, nitro-PAHs, and endotoxins, critically influence immune interactions in dictating how PM interacts with immune cells [87,89]. For instance, PAHs and nitro-PAHs are known to activate the AhR pathway, which can alter immune cell function and promote inflammatory responses [158]. Similarly, heavy metals like lead, cadmium, and arsenic can disrupt cellular signaling pathways, impairing immune regulation and contributing to systemic inflammation [159]. The combination of PM’s physical properties and its xenobiotic toxicant load amplifies its potential to trigger or worsen immune-related diseases, highlighting the need for comprehensive strategies to address both PM emissions and the toxic substances they carry.

#### 3.2.1. Activation of Innate Immunity and Chronic Inflammation

PM primarily activates the innate immune system, with alveolar macrophages, neutrophils, and dendritic cells (DCs) detecting PM components via pattern recognition receptors, such as TLRs [176,177]. This triggers the release of pro-inflammatory cytokines (e.g., IL-6, TNF-α, IL-1β) and chemokines, initiating acute inflammation. Chronic exposure sustains immune activation, leading to tissue damage and diseases like asthma, COPD, and lung fibrosis [178,179]. The PM composition also modulates immune responses. For example, endotoxins activate TLRs, while metals like Fe and Ni catalyze ROS formation, amplifying inflammation and oxidative stress [180]. PAHs and nitro-PAHs interact with aryl hydrocarbon receptors, altering DC function and promoting autoimmunity and T helper 2 cell (Th2)-skewed responses [181].

#### 3.2.2. Disruption of Adaptive Immune Responses

PM also affects adaptive immunity by altering T helper cell subsets (Th1, Th2, Th17) and regulatory T cells (Tregs). PAHs promote Th2-skewed responses, exacerbating allergic inflammation and asthma, while metals like Cd drive Th17 responses linked to autoimmune diseases [164,182,183]. PM may impair Treg function, reducing immune tolerance and promoting chronic inflammation. Diesel exhaust particles enhance IgE production, contributing to allergic sensitization, while PM disrupts DC antigen presentation, weakening adaptive immunity [184,185,186].

#### 3.2.3. Immunosuppression and Impaired Host Defense Mechanisms

Despite its pro-inflammatory effects, PM can suppress immune functions, impairing macrophage phagocytosis and NK cell cytotoxicity, which reduces pathogen clearance and increases the infection risk [187,188,189]. These effects are pronounced with PM containing organic toxicants or metals, leading to increased susceptibility to respiratory infections (e.g., influenza, pneumonia, COVID-19), reduced vaccine efficacy as well as heightened vulnerability to emerging pathogens [190,191].

#### 3.2.4. Autoimmune Disorders and Neuroinflammation

Chronic PM exposure is linked to autoimmune diseases like rheumatoid arthritis and systemic lupus erythematosus, driven by metal and organic components that act as adjuvants, enhancing immune responses to self-antigens and promoting oxidative stress [192]. UFP can translocate into systemic circulation and the central nervous system (CNS), where it activates microglia, inducing neuroinflammation associated with cognitive decline and neurodegenerative diseases such as Alzheimer’s and Parkinson’s [42,193].

While evidence of PM-induced immunotoxicity is constantly growing, the exact mechanisms underlying the cellular uptake, translocation, and distribution of PM components still remain poorly understood. Investigating these pathways in relevant cell types will therefore provide critical insights into PM internalization and trafficking, guiding therapeutic and preventive strategies to mitigate its health impacts.

### 3.3. Impact of PM on the Respiratory System

When PM is inhaled, it deposits in the respiratory tract, including the alveoli and bronchioles. The size and composition of the particles determine their deposition site within the lungs, frequently triggering an immune response [194,195]. Respiratory diseases such as asthma and COPD are significantly impacted by chronic inflammation upon PM exposure [9,196,197]. PM can therefore exacerbate asthma symptoms and increase the frequency of asthma attacks, as well as contribute to the progression of COPD by inducing inflammation and oxidative stress in the respiratory tract [197]. PM_2.5_ and UFP can penetrate deep into the lungs, where these particles disrupt macrophage function, including the production of TNF-α, which is a critical cytokine involved in granuloma formation [198]. The associated granuloma formation, pulmonary fibrosis, and mucous hyperplasia compromise the immune response, potentially leading to more severe manifestation of pulmonary diseases [199,200,201]. As a consequence, these results also suggest that the more PM_2.5_ is inhaled in the lungs, the greater the potential risk to lung health becomes [202,203].

In addition to the effects described, xenobiotic toxicants bound to PM can elicit a range of additional health impacts by amplifying the toxicological burden on the respiratory system, for example, interference with cellular signaling pathways through heavy metal compounds, impairing the repair mechanisms of lung tissue and promoting chronic inflammation [204]. These metals can also induce DNA damage, increasing the risk of lung cancer and other malignancies [205]. Similarly, PAHs and nitro-PAHs, which are common components of PM, can generate ROS and oxidative stress, leading to lipid peroxidation, protein damage, and mitochondrial dysfunction in lung cells [206]. This could worsen conditions like asthma and COPD by enhancing airway hyperresponsiveness or impairing the clearance of pathogens, thereby increasing susceptibility to respiratory infections.

### 3.4. Impact of PM on the Cardiovascular System

PM can penetrate deep into the lungs and enter the bloodstream, triggering systemic inflammation, oxidative stress, as well as endothelial dysfunction, and thereby poses significant dangers also to the cells and tissues of the cardiovascular system. Since PM exposure is associated with chronic inflammation, the release of inflammatory cytokines and chemokines can cause tissue damage and remodeling. After these inflammatory mediators have entered the bloodstream, they can lead to systemic inflammation and oxidative stress, also affecting the cardiovascular system. PM can therefore lead to heart attacks, hypertension, and other cardiovascular conditions by promoting systemic inflammation, oxidative stress, and endothelial dysfunction [207,208,209]. Such processes can also ultimately lead to the development or exacerbation of conditions like hypertension, atherosclerosis, and arrhythmias, and long-term exposure to PM has already been linked to an increased risk of heart attack, stroke, and cardiovascular mortality [210].

Chronic inflammation also promotes the formation of plaques in the arteries, initiating the formation of atherosclerosis [211]. The characteristic plaques consist of lipids, immune cells, and fibrous tissue, also increasing the overall risk of heart attack and stroke [212]. Furthermore, inflammatory cytokines can enhance the coagulation cascade, leading to an increased risk of thrombotic blood clot formation [213]. The adverse cardiovascular effects of PM exposure are particularly pronounced in vulnerable populations such as the elderly, individuals with pre-existing cardiovascular conditions, and those with compromised immune systems. These groups may have heightened susceptibility to the harmful effects of PM-induced inflammation and oxidative stress [214].

In addition to the cardiovascular effects described, toxic xenobiotic substances bound to PM can further exacerbate these health risks by introducing additional mechanisms of toxicity. For example, heavy metals such as cadmium, lead, and arsenic can interfere with calcium signaling and ion channel function, potentially contributing to arrhythmias and impaired cardiac contractility [215,216]. Xenobiotic toxicants may also amplify the pro-thrombotic effects of PM exposure. For instance, certain metals and organic compounds can enhance platelet activation and aggregation, increasing the risk of thrombus formation and subsequent cardiovascular events such as heart attacks and strokes [217,218]. Additionally, these substances may disrupt lipid metabolism, leading to the accumulation of oxidized low-density lipoprotein (LDL) in arterial walls, which accelerates the development of atherosclerotic plaques [219].

### 3.5. Reproductive and Neurodevelopmental Effects of PM Exposure

Systemic inflammation and oxidative stress elicited by PM exposure can also affect placental function, impacting the tissue crucial for nutrient and oxygen exchange between the mother and the developing fetus [220,221]. Inflammation can impair placental blood flow and nutrient transport, ultimately leading to fetal growth restriction since the developing organs and systems of the fetus are highly sensitive to environmental insults. Furthermore, exposure to inflammatory mediators can disrupt normal developmental processes, leading to structural and functional abnormalities [222]. The reproductive and developmental effects of PM exposure that have been described include birth defects and low birth weight [223]. PM can affect fetal development by disrupting placental function and inducing oxidative stress and inflammation, linking maternal exposure to PM during pregnancy to adverse birth outcomes [224,225].

PM exposure during early periods of brain development is especially critical since it might increase the risk of the development of neurodevelopmental problems such as autism spectrum disorder (ASD) and attention-deficit/hyperactivity disorder (ADHD) [226,227,228]. Prenatal and early-life exposure to air pollutants, particularly PM_2.5_ and UFP, nitrogen dioxide, and PAHs, has been associated with an increased risk of ASD [229]. These pollutants can cross the placental barrier or enter the developing brain, leading to neuroinflammation, oxidative stress, and disruptions to neural development [230]. Additionally, air pollution may interfere with critical signaling pathways and epigenetic mechanisms that regulate brain function during early development [230]. While the exact biological mechanisms remain under investigation, the growing body of evidence underscores the importance of reducing air pollution exposure during pregnancy and early childhood to mitigate potential neurodevelopmental risks.

Later in life, the neurological effects induced by PM exposure include cognitive decline and the development of neurodegenerative diseases such as Alzheimer’s and Parkinson’s disease [231,232]. The chronic inflammation and oxidative stress accelerate the pathological processes underlying these conditions. Systemic inflammation and oxidative stress can compromise the integrity of the blood–brain barrier, which normally protects the brain from harmful substances. This disruption allows inflammatory mediators and potentially PM itself to enter the CNS and induce neuroinflammation, oxidative stress, and neuronal damage [233].

Once inside the CNS, PM and inflammatory mediators can activate the microglia, the resident immune cells of the brain. Activated microglia release pro-inflammatory cytokines and ROS, leading to a process known as neuroinflammation [234]. Such chronic neuroinflammation and oxidative stress can in the long term disrupt normal neuronal function, leading to synaptic dysfunction, impaired neurotransmission, and neuronal loss [235]. This can ultimately contribute to conditions such as mood disorders, depression or mild cognitive impairment, and it is also linked to an increased risk of the development of neurodegenerative diseases such as Alzheimer’s disease and Parkinson’s disease [236].

Other studies found elevated levels of TNF-α, the 42-residue amyloid β peptide, and tau hyperphosphorylation on serine 199 in both the temporal and frontal lobes of rats undergoing sub-chronic exposure to diesel exhaust, indicating the upregulation of Alzheimer’s disease markers [237,238]. Additionally, the synuclein levels were elevated in children and young adults, suggesting that prolonged exposure to air pollution might be associated with such an early Parkinson’s disease-like pathology [239]. Another study also noted an increase in TNF-α levels over time, while the response of other pro-inflammatory factors was not consistent, implying that longer exposure to air pollution might trigger a compensatory response to neuroinflammation in the midbrain [240].

Persistent organic pollutants (POPs) and PAHs bound to PM can also disrupt placental function by interfering with trophoblast differentiation and invasion, processes essential for proper placental development and function. These substances may impair the production of key placental hormones, such as human chorionic gonadotropin (hCG) and placental growth factor (PlGF), further compromising fetal growth and increasing the risk of preterm birth or low birth weight [241]. Later in life, xenobiotic toxicant substances may amplify the risk of neurodegenerative diseases by promoting protein misfolding and aggregation [242]. For example, heavy metals like cadmium and lead can enhance the aggregation of amyloid-β and tau proteins, key pathological markers of Alzheimer’s disease, while also impairing the clearance of these proteins by the brain’s glymphatic system [243]. Similarly, exposure to PAHs and other organic pollutants may exacerbate α-synuclein aggregation, a hallmark of Parkinson’s disease, by inducing oxidative stress and mitochondrial dysfunction in dopaminergic neurons [244,245].

### 3.6. Genotoxic and Carcinogenic Effects of PM

Genotoxicity and carcinogenicity are also significant concerns following exposure to pollutants from the environment through various modes of action (Table 5). Genotoxicity refers to the ability to cause damage to the genetic material within cells, leading to mutations and chromosomal aberrations. Since PM exposure has been reported to indirectly cause damage to DNA, chronic exposure to PM can potentially contribute to the development of various forms of cancer [246]. In addition, the direct interaction of PM with biological molecules, such as proteins and DNA, can also disrupt normal cellular functions and promote carcinogenesis [247].

The various harmful components that can be contained in PM can interact with DNA directly or indirectly. PAHs can be metabolized into reactive intermediates that form DNA adducts, leading to mutations during DNA replication [248]. Heavy metals like As and Cd can interfere with DNA repair mechanisms, increasing the likelihood of mutations [13]. Additionally, PM-induced oxidative stress generates ROS that can cause oxidative damage to DNA, resulting in strand breaks and base modifications [249]. This DNA damage can activate signaling pathways that lead to cell cycle arrest, apoptosis, or uncontrolled cell proliferation if the damage is not properly repaired [4,250,251]. Chronic exposure to PM and persistent DNA damage can therefore result in the accumulation of mutations, which may contribute to the initiation and progression of various malignancies. The inflammatory response triggered by PM exposure has been convincingly linked to cancer development, as chronic inflammation can create a microenvironment that supports tumor growth and progression [252,253].

Another potential mechanism involves the disruption of the cellular redox balance by xenobiotic toxicants. For example, heavy metals and organic pollutants can deplete cellular antioxidants such as glutathione, exacerbating oxidative stress and increasing the production of ROS [254]. This heightened oxidative environment not only damages DNA but also promotes lipid peroxidation and protein oxidation, which can further disrupt cellular signaling pathways and contribute to carcinogenesis [255].

**Table 5:** Mechanisms of action of carcinogenic substances bound to particulate matter.

Mode of Action	Description	Examples of Relevant Substances
**Oxidative Stress**	Generation of ROS leads to DNA damage, protein oxidation, and lipid peroxidation, promoting mutations and genomic instability [134].	Arsenic (As) [256].
**DNA Damage and Mutagenesis**	Direct damage to DNA, such as strand breaks, cross-linking, or formation of DNA adducts, resulting in mutations that initiate carcinogenesis [257].	Hexavalent Chromium (Cr(VI)), Benzo[a]pyrene (a PAH) [258,259].
**Chronic Inflammation**	Persistent activation of immune cells and release of pro-inflammatory cytokines generate ROS and reactive nitrogen species, indirectly damaging DNA [260].	Crystalline Silica [261].
**Epigenetic Alterations**	Changes in DNA methylation, histone modification, or microRNA expression alter gene regulation, silencing tumor suppressor genes or activating oncogenes [262].	Arsenic (As), Cadmium (Cd) [263,264].
**Disruption of Cellular Signaling Pathways**	Interference with pathways regulating cell proliferation, apoptosis, and DNA repair leads to uncontrolled cell growth and resistance to cell death [265].	Cadmium (Cd), Lead (Pb) [216,266].

ROS, reactive oxygen species.

### 3.7. Influence of PM on the Human Microbiome

PM and xenobiotic toxicant chemicals have been shown to significantly impact the human microbiome of the respiratory tract as well as the gastrointestinal tract system through mechanisms such as inflammation, oxidative stress, and gut barrier dysfunction (Table 6) [267,268,269]. However, xenobiotic toxicants primarily act through chemical toxicity and interference with microbial metabolism, while particulate matter exerts its effects through physical interactions and the delivery of adsorbed toxins. These disruptions can lead to dysbiosis, increased intestinal permeability compromising the intestinal barrier and promoting the translocation of harmful substances into the bloodstream, and the development of diseases such as inflammatory bowel disease, metabolic disorders, and systemic inflammation [270]. When inhaled, PM and xenobiotic toxicant chemicals can alter the composition and diversity of the respiratory microbiome, leading to dysbiosis—a state of microbial imbalance [271]. This dysbiosis can weaken the immune defenses of the respiratory tract, increasing susceptibility to infections, inflammation, and chronic conditions such as asthma and COPD [272]. These changes in the gut microbiota have been linked to a range of pathophysiological consequences, including metabolic disorders, cardiovascular diseases, and neuroinflammatory conditions, and even were associated with an increased risk of early-onset Crohn disease [273,274].

Another potential mechanism involves the impact of xenobiotic toxicant substances on the gut–brain axis, the bidirectional communication network between the gut microbiome and the central nervous system. Dysbiosis induced by toxins can lead to the production of neuroactive metabolites, such as lipopolysaccharides (LPSs) and microbial-derived tryptophan metabolites, which can cross the blood–brain barrier and contribute to neuroinflammation, mood disorders, and neurodegenerative diseases [275]. For example, elevated levels of LPSs in the bloodstream, resulting from increased intestinal permeability, have been linked to systemic inflammation and neuroinflammatory conditions such as Alzheimer’s disease and Parkinson’s disease [276].

Recent studies utilizing whole-genome sequencing approaches, as well as a comprehensive examination of the literature, also suggest that exposure to air pollutants may increase the risk of obesity and type 2 diabetes through alterations to the gut microbiome [277,278,279].

**Table 6:** Impact of xenobiotic toxicant substances and particulate matter on microbial composition, inflammation, and host–microbe interactions.

	Xenobiotic Toxicant Substances	Particulate Matter
**Disruption of Microbial Composition**	Chemicals or heavy metals can selectively inhibit beneficial microbes and promote pathogens (e.g., *Pseudomonas aeruginosa*) [280,281].	Can act as a physical carrier for pathogenic microbes introducing new microbes and shifting microbial balance toward pro-inflammatory species [282].
**Induction of Inflammation**	Directly activates immune cells, leading to cytokine release (e.g., IL-6, IL-8, and TNF-α), ROS generation and dysbiosis [283].	Activates pattern recognition receptors (e.g., TLRs), inducing ROS and inflammatory cytokines; exacerbates oxidative stress via physical interaction with epithelial cells [284].
**Impairment of Epithelial Function**	Reduces ciliary function in the lung and mucus production, allowing microbial overgrowth; promoting epithelial integrity in the gastrointestinal system (“leaky gut” syndrome) [285,286].	Physically obstructs cilia and increases mucus viscosity, impairing clearance in the lung; physically disrupts the mucus layer and weakens tight junctions in the gastrointestinal tract [287,288].
**Alterations of Host–Microbe Interactions**	Reduces immune defenses, promoting pathogen colonization [289].	Acts as an adjuvant, hyperactivating immune responses and disrupting microbial homeostasis [173,290].

IL, interleukin; TNF, tumor necrosis factor; ROS, reactive oxygen species; TLR, toll-like receptor.

### 3.8. Systemic Risk Modeling

Advances in computational toxicology and integrated risk assessment tools have revolutionized the ability to evaluate the health risks associated with PM and its adsorbed toxicants [291]. Computational toxicology leverages in silico models to predict the biological effects of chemical exposures, reducing reliance on traditional in vivo and in vitro methods [292]. Tools such as the Stochastic Human Exposure and Dose Simulation (SHEDS) model enable detailed exposure assessments by simulating human contact with environmental contaminants across various pathways and time scales [293]. Similarly, physiologically based pharmacokinetic (PBPK) models provide a mechanistic framework for predicting the absorption, distribution, metabolism, and excretion of chemicals within the body, offering insights into dose–response relationships and interindividual variability [294,295]. Exposome-based approaches further enhance risk assessment by integrating data on the totality of environmental exposures over a lifetime, including PM and its adsorbed components, with biological responses such as gene expression and metabolomics [296]. These tools are particularly valuable for assessing the risks posed by PM-bound toxicants, such as PAHs, heavy metals, and persistent organic pollutants, which can exacerbate the health impacts of PM exposure. By combining computational models with high-throughput data from omics technologies, researchers can identify critical exposure pathways, vulnerable populations, and potential intervention points. Moreover, these approaches facilitate the development of predictive models that account for the complex interactions between PM, co-pollutants, and host factors, ultimately supporting more accurate and comprehensive risk assessments [297]. As these tools continue to evolve, their integration into regulatory frameworks and public health strategies will be essential for mitigating the health risks associated with PM exposure.

A deeper understanding of source-specific PM toxicity is critical for designing targeted interventions to reduce health risks and inform actionable policy measures. Different PM sources, such as vehicular emissions, industrial activities, biomass burning, and secondary aerosols, contribute distinct chemical compositions and toxicological profiles [298]. For instance, PM from traffic emissions is often enriched with black carbon and PAHs, which are associated with oxidative stress and cardiopulmonary diseases, while biomass burning releases fine particles with high organic carbon content, linked to respiratory inflammation [299,300]. Source apportionment studies using techniques like receptor modeling, isotopic analysis, and chemical transport models can help identify the most harmful sources and their contributions to ambient PM levels [301]. These insights can guide policy measures, such as stricter emission standards for vehicles, promotion of cleaner industrial technologies, and incentives for renewable energy adoption. Urban areas, in particular, require targeted strategies to mitigate emissions from key sources, including the expansion of public transportation, implementation of low-emission zones, and regulation of construction dust. By focusing on source-specific toxicity and integrating these findings into health burden assessments, policymakers can prioritize interventions that yield the greatest public health benefits.

## 4. Strategies for Mitigating PM Exposure

Mitigation strategies for addressing PM exposure are essential to safeguard public health and improve air quality. Addressing these challenges requires a multifaceted approach that combines technological, regulatory, and behavioral interventions. Effective mitigation strategies should ideally focus on reducing PM emissions at their source but should also include enhancing air quality monitoring systems and promoting public awareness about protective measures. By implementing these strategies, governments, industries, and individuals can work together to minimize exposure to harmful particulate matter and create healthier living environments.

### 4.1. Regulatory Policies and Standards for PM Emission Control

Established standards represent critical tools in the fight against air pollution and its harmful effects on public health and the environment. An outstanding example in this respect is the Environmental Protection Agency (EPA), which has established regulations on the limits of PM concentrations in the air to protect public health. Regulatory frameworks, such as the Clean Air Act in the United States or the European Union’s Air Quality Standards, provide guidelines for monitoring, reporting, and enforcing compliance [302]. The EPA’s National Ambient Air Quality Standards (NAAQS) specify the permissible levels of PM_10_ and PM_2.5_, aiming to reduce exposure and the associated health risks [303].

In addition to setting concentration limits, regulatory policies increasingly emphasize the adoption of advanced technologies and best practices for particulate matter (PM) emission control at the source (see also Section 4.2). Furthermore, policies are evolving to address non-industrial sources of PM, such as emissions from transportation, residential heating, and agriculture [304,305]. Measures like promoting the use of electric vehicles, transitioning to cleaner fuels, and incentivizing sustainable agricultural practices are becoming integral components of comprehensive PM control strategies [306]. These proactive approaches will eventually not only help in meeting healthy air quality standards but also contribute to long-term sustainability goals by reducing the overall environmental footprint.

### 4.2. Technological Innovations in Emission Reduction

Recent advancements have become a cornerstone of the global effort to mitigate exposure to PM. Approaches in this field focus on reducing PM emissions from key sources such as industrial processes, transportation, and energy production. Innovations such as advanced filtration systems, catalytic converters, and cleaner fuel technologies have significantly reduced PM emissions from industrial processes and vehicles [307]. Such cutting-edge technologies are designed to capture and neutralize fine particles before they are released into the atmosphere. Additionally, the integration of real-time monitoring systems has enhanced the efficiency of emission control by enabling predictive maintenance and optimizing operational processes. These advancements are also necessary to help industries to comply with increasingly stringent environmental regulations. As technology continues to evolve, it therefore holds immense potential to further reduce PM emissions and address the challenges of air pollution on a global scale.

Another promising area of innovation in emission reduction is the development and deployment of nature-based and bio-inspired technologies. For example, biofiltration systems, which use living microorganisms to capture and degrade particulate matter and other pollutants, are gaining traction as sustainable alternatives to traditional filtration methods [308]. Similarly, urban greening initiatives, such as the strategic planting of trees and vegetation, have been shown to naturally filter airborne particles while providing additional benefits like carbon sequestration and urban cooling [309,310]. Emerging research is also exploring the use of nanotechnology to create advanced materials capable of capturing ultrafine particles with unprecedented efficiency [311]. These approaches could complement existing technological solutions and also align with broader environmental goals by promoting ecosystem health and reducing reliance on energy-intensive processes.

### 4.3. Approaches to Maintaining High Air Quality

This might represent one of the most critical factors in maintaining human health. The development of specialized filtration systems for different settings—including homes, workplaces, schools, and healthcare facilities—has emerged as a vital tool in mitigating health risks by removing particulate matter from the air [312,313,314,315]. High-efficiency particulate air (HEPA) filters have already been developed as a useful tool to remove PM, hence improving indoor air quality [316]. Other studies investigated the effects of portable air filtration systems on the exposure to PM and the correlation to blood pressure among residents of a low-income senior facility [317]. A comprehensive review dealt with various individual- and household-level interventions to reduce air pollution exposures and minimize the associated health risks [318]. The Reducing Air Pollution in Detroit Intervention Study was designed to evaluate the cardiovascular health benefits and personal fine particulate matter (PM_2.5_) exposure reductions achieved via portable air filtration units among older adults in Detroit, Michigan. It was also shown that commercially available HEPA-type and true HEPA personal air filters mitigated the median indoor PM_2.5_ concentrations by 58% and 65%, respectively [319].

### 4.4. Degradation of Xenobiotic Pollutants

Recent technological progress achieved in this area might represent the most effective and useful metabolic approach to degrading xenobiotic compounds like azo dyes, phenolics, PAHs, halogenated compounds, pesticides, nitroaromatic compounds, triazines, and chlorinated compounds [320,321]. Xenobiotic toxicant substances represent a major environmental pollution threat originating from both natural and human-made processes. The ability of different microorganisms to survive and thrive in the most diverse environments and also produce enzymes and metabolites that break down and inactivate pollutants could facilitate contaminated areas recovering naturally [322,323,324]. Additionally, emerging technologies like nanotechnology and photocatalysis are being explored to enhance the efficiency of degradation processes [325]. Genetic engineering of microbes to target specific xenobiotics and the development of bioaugmentation strategies further expand the potential for effective pollutant removal [326,327]. By harnessing these innovative approaches, it could eventually become possible to mitigate the long-term environmental and health impacts of xenobiotic pollutants, paving the way for cleaner and more sustainable ecosystems.

### 4.5. Role of Nutrition in Mitigating PM Effects

Components that are effective in countering the toxic effects of chemical contaminations in food include antioxidants, phytochemicals, essential fatty acids, and detoxification-supporting compounds. Together, nutritional components can work synergistically to support the protection of the body from the harmful effects of chemical contaminants in food. Antioxidants such as vitamins C and E, as well as selenium in the form of selenoproteins, play a crucial role in neutralizing ROS generated by chemical toxins, thereby reducing oxidative stress and preventing cellular damage [328]. Also, phytochemicals like flavonoids, carotenoids, and polyphenols, found in fruits, vegetables, and green tea, exhibit strong free-radical-scavenging properties and modulate detoxification pathways [329]. Sulfur-containing compounds, such as those found in garlic, onions, and cruciferous vegetables, enhance the activity of phase II detoxification enzymes in the liver, facilitating the conjugation and excretion of harmful xenobiotics [330]. Omega-3 fatty acids, present in fatty fish, flaxseeds, and walnuts, have anti-inflammatory properties that help mitigate inflammation caused by chemical contaminants [331]. Additionally, dietary fiber, found in whole grains, legumes, and fruits, binds to toxins in the gastrointestinal tract, promoting their elimination and reducing their absorption into the bloodstream [332]. Minerals also play a vital role in the detoxification processes of the human body by supporting various enzymatic functions. For example, Zn and Mg support enzymatic functions involved in liver detoxification pathways, particularly in neutralizing free radicals and repairing damaged tissues [333]. Se acts as a cofactor for glutathione peroxidase, a key antioxidant enzyme that helps eliminate harmful peroxides generated during detoxification [334]. Additionally, Fe and Cu are involved in the metabolism of toxins and the synthesis of detoxifying enzymes [335,336].

### 4.6. Applications of Artificial Intelligence in PM Management

The use of artificial intelligence (AI) will also prove helpful in the near future by playing a pivotal role in predicting potentially dangerous exposure to PM to minimize negative health effects for people or populations at risk. By leveraging machine learning algorithms and large datasets, AI has been already been described to analyze historical air quality data, meteorological conditions, and real-time sensor readings to forecast PM levels with high accuracy [337]. These predictions can help to identify high-risk areas and times, enabling authorities to issue timely warnings to the public. AI can also integrate satellite imagery and traffic data to pinpoint the sources of pollution, such as industrial emissions or vehicular traffic, and assess their impact on air quality [338]. Potential solutions include deploying AI-powered air quality monitoring systems in urban and industrial areas, optimizing traffic flows to reduce emissions, and implementing predictive maintenance for industrial equipment to minimize pollutant release. Additionally, AI can assist in designing personalized health alerts for vulnerable populations, such as children, the elderly, and individuals with respiratory conditions, advising them to take precautions during periods of high PM exposure [339]. Such public health interventions and awareness programs could prove essential for educating communities about the risks of PM exposure and promoting behaviors that reduce exposure. Initiatives such as air quality monitoring, public advisories, and community engagement can help individuals take protective measures. Future research should focus on understanding the long-term health effects of PM exposure and developing more effective mitigation strategies.

Besides systemic risk modeling, the advancements in AI offer transformative opportunities to address the complex interplay between PM exposure, health outcomes, and policy interventions. AI-driven tools, such as digital twins, enable the creation of virtual replicas of urban environments, allowing researchers to simulate the impacts of various emission reduction strategies on air quality and public health in real time [340]. Smart health early warning systems, powered by machine learning algorithms, can integrate air quality data with health surveillance systems to predict and mitigate acute health events, such as asthma exacerbations or cardiovascular emergencies, during high PM episodes [341,342]. Integrative frameworks that combine omics technologies (e.g., genomics, proteomics, and metabolomics), exposure modeling, and policy analysis provide a holistic approach to understanding PM-related health risks. For example, exposome-based frameworks can link molecular-level changes caused by PM exposure to population-level health outcomes, offering insights into both biological mechanisms and societal impacts [343]. By bridging the gap between scientific research, technological innovation, and policy implementation, these forward-looking approaches can drive more effective and equitable solutions to the challenges posed by PM pollution.

## 5. Conclusions

PM exposure poses significant health concerns and represents a potential epidemiological threat to society. The key findings indicate that PM exposure is associated with a range of adverse health effects, including respiratory diseases such as asthma and COPD, cardiovascular diseases like heart attack and hypertension, neurological effects such as cognitive decline and neurodegenerative diseases, and reproductive and developmental issues, including birth defects and low birth weight. These observations highlight the risks of potential epidemiological threats, particularly due to the effects of xenobiotic toxicants often associated with PM. These substances, such as heavy metals, PAHs, and VOCs, exacerbate the harmful effects of PM by amplifying oxidative stress, inflammation and systemic toxicity. These effects ultimately contribute to increased morbidity and mortality rates, particularly among vulnerable populations such as the elderly, children, and individuals with pre-existing health conditions.

PM pollution remains a pressing global challenge, with profound implications for public health, environmental sustainability, and social equity. The financial burden on the healthcare system can also be substantial, as treating PM-related illnesses requires significant medical resources and leads to higher healthcare costs. Addressing the multifaceted risks associated with PM exposure therefore requires a paradigm shift toward integrative frameworks that combine advanced scientific methodologies, predictive modeling, and evidence-based policy interventions. Omics technologies, such as genomics, proteomics, and metabolomics, offer unprecedented insights into the molecular mechanisms underlying PM-induced health effects, enabling the identification of biomarkers for exposure and susceptibility. When coupled with exposure modeling tools, such as land-use regression, satellite-based monitoring, and computational toxicology models, these approaches provide a comprehensive understanding of the pathways linking PM sources to health outcomes. Furthermore, the integration of these scientific advancements into policy frameworks is essential for translating research findings into actionable solutions. Policies informed by source-specific toxicity data, systemic risk modeling, and environmental justice considerations can prioritize interventions that maximize public health benefits while addressing disparities in exposure and vulnerability. By fostering collaboration across disciplines and sectors, integrative frameworks can bridge the gap between science and policy, driving innovative strategies to mitigate PM pollution and protect human health. This holistic approach is not only critical for managing current challenges but also for building resilience against future environmental and public health crises.

The importance of continued research and policy development to reduce PM exposure cannot be overstated. Effective regulatory policies, such as the EPA’s NAAQS, are crucial for setting limits on PM concentrations and protecting public health. Technological advancements in emission control, including advanced filtration systems and cleaner fuel technologies, play a vital role in reducing PM emissions. Public health interventions and awareness programs are essential for educating communities and promoting protective behaviors. Future research should focus on understanding the long-term health effects of PM exposure, developing more effective mitigation strategies, and assessing the economic benefits of reducing PM levels.

## Figures and Tables

**Table 1:** Comparison of classical xenobiotic toxicants and particulate matter: nature, composition, exposure, and health impacts.

	Classical Xenobiotic Toxicants	Particulate Matter
**Nature and Origin**	Chemicals not naturally produced by the body or commonly found in the environment (e.g., pesticides, industrial chemicals, and pharmaceuticals) [17,18,19].	Complex mixture of tiny particles and liquid droplets suspended in the air, originating from both natural sources (e.g., sand, dust, pollen, sea salts) and anthropogenic sources (e.g., emissions from vehicles, industrial activities) [20,21].
**Composition**	Specific chemical substances with defined molecular structures [22].	Often contains a variety of substances, including xenobiotic and toxic pollutants like heavy metals and organic compounds; particles themselves are not necessarily toxic but exert a variety of physiological and pathophysiological effects [13].
**Health Effects**	Directly cause adverse health effects due to their chemical properties and interactions with biological systems [23,24].	Exerts effects through mechanisms such as the generation of ROS and oxidative stress, leading to cellular damage, inflammation, and disruption of normal cellular functions [25,26].
**Exposure Pathways**	Typically enter the body through ingestion, inhalation, or dermal absorption [27,28,29].	Primarily enters the body through inhalation and ingestion, depositing in the respiratory system and the gastrointestinal tract and potentially causing systemic effects [30,31].
**Health Outcomes**	Associated with specific toxicological effects depending on the substance (e.g., neurotoxicity, carcinogenicity) [32,33].	Linked to a broad range of health issues, including respiratory diseases (e.g., asthma, COPD), cardiovascular diseases (e.g., heart attacks, hypertension), neurological effects (e.g., cognitive decline, neurodegenerative diseases), and reproductive and developmental issues (e.g., birth defects, low birth weight) [34,35,36].

ROS, reactive oxygen species; COPD, chronic obstructive pulmonary disease.

**Table 2:** Toxicokinetics and toxicodynamics of key compounds in particulate matter: mechanisms of absorption, distribution, and potential health effects.

Compound	Toxicokinetics	Toxicodynamics
**Heavy Metals (e.g., Pb, Cd, As)**	Soluble metals can cross biological barriers (e.g., alveolar–capillary membrane) and enter the bloodstream, leading to systemic distribution; may accumulate in organs such as the liver and kidneys [88,89].	Can interfere with cellular processes by binding to proteins and enzymes; induces oxidative stress and inflammation, contributing to organ damage and chronic diseases [90].
**Transition Metals (e.g., Fe, Cu, Ni)**	Catalyze the production of ROS via Fenton-like reactions; may remain in lung tissues, prolonging exposure [91].	ROS generation leads to oxidative damage to lipids, proteins, and DNA; contributes to respiratory and cardiovascular diseases [91].
**Polycyclic Aromatic Hydrocarbons (PAHs)**	Lipophilic nature allows absorption through the respiratory tract and distribution into fatty tissues; metabolized in the liver via cytochrome P450 enzymes, forming reactive intermediates [92].	Reactive intermediates can form DNA adducts, leading to mutations and increased cancer risk; induces oxidative stress and inflammation [93].
**Nitro-PAHs**	Similar to PAHs, nitro-PAHs are metabolized into reactive intermediates; can persist in tissues due to their chemical stability.	Forms DNA adducts, increasing genotoxicity and cancer risk; potent inducers of oxidative stress [94].
**Endotoxins (Biological Sources)**	Endotoxins are not absorbed systemically but remain in the respiratory tract, where they interact with immune cells.	Activates TLRs on immune cells, triggering inflammation and release of pro-inflammatory cytokines; exacerbates respiratory diseases like asthma and COPD [95].
**Soluble Metals (e.g., Ni, V)**	Soluble metals are absorbed into the bloodstream and distributed systemically; can accumulate in cardiovascular tissues.	Causes systemic inflammation, endothelial dysfunction, and increased blood coagulation; contributes to cardiovascular diseases [96].
**Neurotoxic Metals (e.g., Mn)**	Can cross the blood–brain barrier after systemic absorption; accumulates in brain tissues, leading to prolonged exposure [97].	Induces neuroinflammation and oxidative stress in the brain; associated with neurodegenerative diseases like Parkinson’s and Alzheimer’s [98].

ROS, reactive oxygen species; PAH, polycyclic aromatic hydrocarbon; TLR, toll-like receptor; COPD, chronic obstructive pulmonary disease.

**Table 3:** Mechanisms of interaction between xenobiotic toxicants and particulate matter and implications for distribution, bioavailability, and toxicity.

	Mechanism	Examples
**Adsorption via Van der Waals Forces**	Weak intermolecular forces allow xenobiotic and toxic substances to adhere to the surface of particulate matter.	PAHs adsorbing onto soot particles in urban air pollution [100,101].
**Electrostatic Attraction**	Charged xenobiotic toxicant molecules bind to oppositely charged particulate matter.	Heavy metals like Pb or Cd binding to negatively charged fine dust particles [102].
**Hydrophobic Interactions**	Nonpolar xenobiotic toxicant substances bind to hydrophobic surfaces of particulate matter.	Persistent organic pollutants, such as dioxins, binding to carbonaceous PM [103,104].
**Covalent Bond Formation**	Xenobiotic and toxic substances form covalent bonds with reactive functional groups on particulate matter.	Reactive aldehydes or ketones forming covalent bonds with organic matter in PM [105].
**Physical Entrapment**	Xenobiotic and toxic substances become physically trapped within porous particulate matter.	VOCs trapped in porous volcanic ash or activated carbon particles [106,107].
**Ion Exchange**	Ionic xenobiotic toxicants exchange with ions on the surface of particulate matter.	Ammonium ions (NH_4_^+^) from fertilizers binding to mineral dust particles [108].
**Complexation with Metal Oxides**	Xenobiotic and toxic substances form complexes with metal oxides present on particulate matter.	Forming complexes with iron oxides in PM from mining activities [109].
**Hydrogen Bonding**	Hydrogen bonds form between xenobiotic toxicants and functional groups on particulate matter.	Phenolic compounds binding to hydroxyl groups on silica particles [110].
**Surface Coating**	Xenobiotic and toxic substances coat the surface of particulate matter, forming a thin layer.	Pesticides like DDT coating soil dust particles during agricultural spraying [111,112].
**Aggregation with Organic Matter**	Xenobiotic and toxic substances aggregate with organic matter present in particulate matter.	PCBs binding to organic carbon in PM from industrial emissions [113].

PAH, polycyclic aromatic hydrocarbon; VOC, volatile organic compound; DDT dichlordiphenyltrichlorethan; PCB, polychlorinated biphenyl.

**Table 4:** Interactions between particulate matter and different immune cell types—possible effects on human health and disease.

Immune Cell Type	Type of Interaction	Effects in the Human Body
**Macrophages**	Phagocytosis of particles; activation via pattern recognition receptors (e.g., TLRs) [160].	Release of pro-inflammatory cytokines (e.g., IL-6, TNF-α, IL-1β); ROS generation; NLRP3 inflammasome activation; chronic inflammation and tissue damage in the lungs; cancer metastasis [160,161].
**Neutrophils**	Chemotaxis in response to cytokines; direct activation by particles [162].	Increased neutrophil infiltration, NETosis, ROS release, tissue damage, acute lung injury; exacerbation of respiratory diseases (e.g., asthma, COPD) [163].
**Dendritic Cells (DCs)**	Uptake of particulate matter and presentation of associated antigens; activation by associated components; AhR-mediated signaling [164].	Impaired antigen presentation and T cell activation; skewing of adaptive immune responses (e.g., Th2 or Th17 cell dominance) [165].
**T Helper Cells (Th Cells)**	Skewing of Th cell subsets by associated components; Th2 and Th17 activation by particle-induced cytokines [166].	Th2 dominance promotes allergic inflammation (e.g., asthma); Th17 activation contributes to autoimmune diseases and chronic inflammation [167].
**Regulatory T Cells (Tregs)**	Inhibited induction via tolerogenic DCs; suppression of Treg activity; impaired immune regulation due to oxidative stress and inflammation [168].	Reduced immune tolerance, leading to exacerbation of autoimmune diseases; increased risk of chronic inflammation [169].
**Natural Killer (NK) Cells**	Indirect modulation via cytokine shifts and particle-induced stress [31].	Impaired cytotoxicity; increased susceptibility to infection and tumor growth [170].
**B cells**	Antigen exposure and co-stimulation [157].	Elevated IgE production; exacerbation of allergic responses [157,171].
**Epithelial Cells**	Direct interaction with particles deposited in the respiratory tract; activation via ROS and cytokines [172].	Release of pro-inflammatory mediators (e.g., IL-8, granulocyte–macrophage colony-stimulating factor); recruitment of immune cells to the site of exposure; damage to epithelial barriers, increasing susceptibility to infections [173].
**Microglia (Brain Immune Cells)**	Activation by particles translocated to the brain via the olfactory nerve or systemic circulation [174].	Neuroinflammation and oxidative stress in the brain; linked to neurodegenerative diseases (e.g., Alzheimer’s, Parkinson’s) [175].

TLR, toll-like receptor; IL, interleukin; TNF, tumor necrosis factor; ROS, reactive oxygen species; NLRP, nucleotide-binding domain, leucine-rich-containing family, pyrin domain; COPD, chronic obstructive pulmonary disease; AhR, aryl hydrocarbon receptor; Th, T helper; DC, dendritic cell; Treg, regulatory T cell; NK, natural killer cell.

## Data Availability

No new data were created or analyzed in this study.

## Abbreviations

The following abbreviations are used in this manuscript:

ADHD	Attention-deficit/hyperactivity disorder
AI	Artificial intelligence
ASD	Autism spectrum disorder
CNS	Central nervous system
COPD	Chronic obstructive pulmonary disease
DC	Dendritic cell
DDT	Dichlordiphenyltrichlorethan
EPA	Environmental Protection Agency
HEPA	High-efficiency particulate air
IL-1β	Interleukin-1 beta
IL-6	Interleukin-6
IL-8	Interleukin-8
NAAQS	National Ambient Air Quality Standards
NK	Natural killer cell
PAH	Polycyclic aromatic hydrocarbons
PCB	Polychlorinated biphenyl
PM	Particulate matter
ROS	Reactive oxygen species
Th17	T helper 17 cell
Th2	T helper 2 cell
TLR	Toll-like receptor
TNF-α	Tumor necrosis factor-alpha
Treg	Regulatory T cell
UFP	Ultrafine particle
VOC	Volatile organic compound
